# Using information literacy to teach medical entrepreneurship and health care economics

**DOI:** 10.5195/jmla.2019.577

**Published:** 2019-04-01

**Authors:** Alexander J. Carroll, Shelby J. Hallman, Kelly A. Umstead, James McCall, Andrew J. DiMeo

**Affiliations:** Lead Librarian for Research Engagement, NC State University Libraries, North Carolina State University, Raleigh, NC, ajcarro4@ncsu.edu; Research Librarian for Engineering and Entrepreneurship, Libraries, North Carolina State University, Raleigh, NC, sjhallma@ncsu.edu; Assistant Professor and Director of Graduate Programs, Department of Industrial Design, North Carolina State University, Raleigh, NC, kaumstead@ncsu.edu; Doctoral Student, Department of Biomedical Engineering, Joint University of North Carolina at Chapel Hill and North Carolina State University, Raleigh, NC, jvmccall@ncsu.edu; Innovation and Design Coach, Trig, Chapel Hill, NC, ajdimeo@gmail.com

## Abstract

**Objective:**

Entrepreneurship and innovative product design in health care requires expertise in finding and evaluating diverse types of information from a multitude of sources to accomplish a number of tasks, such as securing regulatory approval, developing a reimbursement strategy, and navigating intellectual property. The authors sought to determine whether an intensive, specialized information literacy training program that introduced undergraduate biomedical engineering students to these concepts would improve the quality of the students’ design projects. We also sought to test whether information literacy training that included active learning exercises would offer increased benefits over training delivered via lectures and if this specialized information literacy training would increase the extent of students’ information use.

**Methods:**

A three-arm cohort study was conducted with a control group and two experimental groups. Mixed methods assessment, including a rubric and citation analysis, was used to evaluate program outcomes by examining authentic artifacts of student learning.

**Results:**

Student design teams that received information literacy training on topics related to medical entrepreneurship and health care economics showed significantly improved performance on aspects of project performance relevant to health care economics over student design teams that did not receive this training. There were no significant differences between teams that engaged in active learning exercises and those that only received training via lectures. Also, there were no significant differences in citation patterns between student teams that did or did not receive specialized information literacy training.

**Conclusions:**

Information literacy training can be used as a method for introducing undergraduate health sciences students to the health care economics aspects of the medical entrepreneurship life cycle, including the US Food and Drug Administration regulatory environment, intellectual property, and medical billing and reimbursement structures.

## INTRODUCTION

Complexities and interdependencies in the US health care system can make necessary and desirable changes complicated, expensive, and slow to implement. Academic medical centers, which provide leadership and direction for the US health care system, have three core service missions: teaching, research, and patient-centered clinical care [1]. Programs and initiatives that deviate from these core functions, including entrepreneurship, can meet resistance due to the scarcity of operating resources in these institutions [2]. Others suggest that the organizational structures and cultures in hospitals, such as lack of individual incentives and poor knowledge management, inhibit innovation [3].

US Food and Drug Administration (FDA) regulations also have a pronounced impact, as inventions and innovations like medical devices can face a lengthy, expensive approval process that may include animal trials and up to three years of human trials. Since the 2010 Affordable Care Act, an increased emphasis on cost-effectiveness has further constricted investments in innovative product commercialization, as reimbursement guidelines now dictate that new products must be cheaper or cost-neutral when compared to existing options that are already on the market [4]. Moreover, securing external funding from industry to sponsor entrepreneurial activities in academic health centers continues to be fraught, as many in health care remain concerned about conflicts of interest that inevitably occur as a result of industry-academic partnerships [5, 6].

Despite these barriers, academic medical centers are uniquely situated to promote innovation in health care, as they benefit from the co-location of patients and health care providers in the same physical spaces as researchers in basic and applied sciences [7]. While clinicians possess critical knowledge for medical innovation, given the demands of patient care, they lack the dedicated time and resources to translate the problems that they encounter in the workplace into commercially viable solutions [8].

Biomedical engineers, who matriculate through an undergraduate curriculum that provides an introduction to the health care environment as well as expertise in design thinking, should be well equipped to address this gap. Biomedical engineers function in academic medical centers as problem solvers and technological entrepreneurs, working with clinicians to design hardware or software solutions that enhance patient care and safety [9]. Beyond their individual contributions, biomedical engineers can provide valuable contributions in team settings by developing solutions that meet the needs of a diverse set of stakeholders, including both clinicians and patients. Carter et al. found that immersive undergraduate research experiences, such as the senior design courses completed by engineering students, can promote development of the communication skills that are necessary to work as a part of interdisciplinary design teams that can include clinicians, engineers, product designers, marketers, and others [10]. Moreover, numerous studies show the benefit of understanding user needs when developing products in the health care environment, which biomedical engineers should be well positioned to learn through the immersive clinical experiences that they often gain in design courses [11–13].

However, biomedical engineers matriculating from undergraduate programs routinely report that they feel unprepared to enter the workforce, suggesting that their current training does not sufficiently prepare them to navigate the complexities of the current health care system [14]. In several preprofessional degree programs, information literacy training has been identified as a way to prepare emerging professionals for the complex tasks that they will encounter in the workplace [15–17]. The health sciences librarianship literature contains numerous examples highlighting the effectiveness of in-person instructional interventions when teaching evidence-based practice skills to health sciences students [18–20]. Likewise, studies in engineering librarianship have demonstrated the effectiveness of embedding information literacy training into the undergraduate curriculum for engineers [21–23]. Scholarship in teaching and learning broadly suggests that training is most effective when content is spread out across several time points and advanced topics are introduced progressively, rather than delivered via a single guest lecture [24, 25].

Undergraduate biomedical engineering (BME) students at North Carolina State University (NC State) are introduced to the medical innovation life cycle through their design sequence courses, which span across their junior and senior years. In each of the junior and senior design courses, students form teams and spend up to two semesters investigating health-related problems and developing working prototypes on a topic of their choice. Through these courses, students are introduced to the Stanford Biodesign Innovation Process, an iterative three-stage process in which medical innovators identify an unmet health need, explore possible solutions, and then implement prototypes [26]. Successfully completing each of these stages requires discovering and synthesizing an array of primary and secondary information sources.

The authors sought to investigate whether information literacy training could be used to improve these undergraduate BME students’ design projects. To measure the effectiveness of introducing an information literacy training intervention in the existing Biodesign curriculum, we developed three hypotheses. We hypothesized that (1) information literacy training that focused on health care economics would improve the quality of student design projects; (2) information literacy training that included active learning exercises would offer increased benefits over training delivered via lectures; and (3) this specialized information literacy training would increase the extent of information that students used.

## METHODS

To prepare students to find information sources during each phase of the Biodesign Innovation Process, a team of librarians developed an instructional intervention that addressed how to find different types of information. Librarians trained students in the junior design course on finding epidemiology data and disease state information, peer-reviewed articles from scholarly journals, patents, and business intelligence information on competitor medical device companies. Librarians introduced students in the senior design course to additional sources that were specifically relevant for medical device development, including standards, legal information, FDA regulatory information, and reimbursement and medical billing information. These resources were collated for students in customized, course-specific web pages on the library website with links from the university’s learning management system [27, 28].

In the first phase of the study, we introduced students to these resources through a librarian-led guest lecture in the junior and senior design courses, each fifty minutes in length. These lectures featured a combination of PowerPoint slides explaining the utility and limitations of different information sources as well as live demonstrations of how to search in different user interfaces. Because our Phase I implementation delivered this intervention via lecture to high-enrollment classes, students did not have time in class to meaningfully explore and practice using these resources in the presence of a librarian.

In the second phase, the instructional intervention for the junior design course remained the same; however, instruction for the senior design course was delivered to four smaller sections in a four-hour lab setting. Gaining additional classroom time with students in their senior design course, combined with the smaller enrollment in each section, created an opportunity to design a more interactive, instructional intervention that featured a number of active learning exercises (supplemental Appendix A and Appendix B). Including these exercises allowed the students to receive hands-on, guided practice time with these concepts and resources, which allowed us to identify and address misunderstandings early in their design process.

To evaluate these interventions and test our 3 hypotheses, we designed a quasi-experimental 3-arm cohort study. The first arm (n=5 student teams, 22 students) was randomly drawn from a sample of student senior design projects that BME students in the class of 2016 completed. Because these projects were completed prior to the implementation of our information literacy training program that emphasized resources related to health care economics, this arm served as this study’s control group. The second and third arms of the study, referred to as “Phase I” (n=5 student teams, 28 students) and “Phase II” (n=7 student teams, 33 students), included a sample of student teams from the BME class of 2017 (Phase I) and class of 2018 (Phase II) [29]. Table 1 provides additional demographic information on each cohort. Notably, the curriculum content for these students did not change substantively during these 3 years, outside of the inclusion of the information literacy intervention. The library instructors delivering the instructional intervention, as well as the instructor of record, remained constant for students in all 3 cohorts.

**Table 1:**
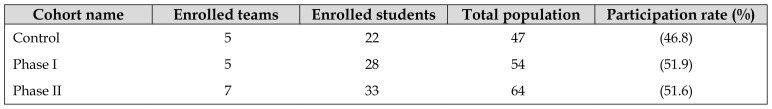
Cohort arm demographics

Cohort name	Enrolled teams	Enrolled students	Total population	Participation rate (%)
Control	5	22	47	(46.8)
Phase I	5	28	54	(51.9)
Phase II	7	33	64	(51.6)

To assess the impact of including information literacy training in the BME design sequence, we created an interdisciplinary research team that included the librarians who delivered the information literacy intervention and the instructor of record for these courses. We utilized a mixed methods approach to test our hypotheses. Because undergraduate engineering students often enter their design projects into design competitions, we designed a rubric based on the evaluation criteria that are used in the National Institute of Biomedical Imaging and Bioengineering/VentureWell Design by Biomedical Undergraduate Teams (DEBUT) Challenge. We evaluated student team performance across four different learning outcomes and used the teams’ total scores on this rubric as an indicator of the quality of their outputs (Table 2 and supplemental Appendix C) [30, 31]. To measure the extent of information that students used, we analyzed cited references in their design project documentation.

**Table 2:**
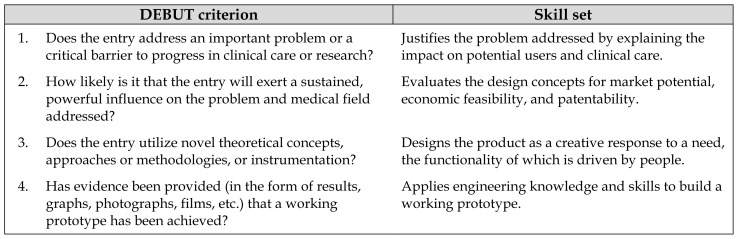
Design by Biomedical Undergraduate Teams (DEBUT) criteria and associated skill sets used to measure student performance

DEBUT criterion	Skill set
1. Does the entry address an important problem or a critical barrier to progress in clinical care or research?	Justifies the problem addressed by explaining the impact on potential users and clinical care.
2. How likely is it that the entry will exert a sustained, powerful influence on the problem and medical field addressed?	Evaluates the design concepts for market potential, economic feasibility, and patentability.
3. Does the entry utilize novel theoretical concepts, approaches or methodologies, or instrumentation?	Designs the product as a creative response to a need, the functionality of which is driven by people.
4. Has evidence been provided (in the form of results, graphs, photographs, films, etc.) that a working prototype has been achieved?	Applies engineering knowledge and skills to build a working prototype.

Our protocol, as approved by NC State’s Institutional Review Board, deemed that student teams in Phase I and Phase II were eligible for inclusion in the study if every member of the team opted into the study via an informed consent document. As such, groups in both phases represent a convenience sample, rather than a random sample. In Phase I, five student teams opted into the study, all of which were included in the final analysis. In Phase II, nine teams opted into the study; however, two of these teams were excluded from our final analysis. One of the excluded teams submitted an oral presentation in lieu of a final written report, while the other team designed a public health awareness campaign rather than creating a medical device prototype. In both cases, we were unable to evaluate these projects accurately using our rubric, which was designed to evaluate written entries that accompany physical prototypes in a design competition.

Members of the interdisciplinary research team individually scored student design projects using the rubric. To promote consistent evaluation, we used randomly drawn student projects from the BME class of 2015 for norming exercises and practiced assigning scores using the rubric. Student projects were assigned a score of 0–10 points for each criterion. The standard deviations (SDs) between individual evaluators’ scores for each criterion across all 3 cohorts are reported in Table 3.

**Table 3:**
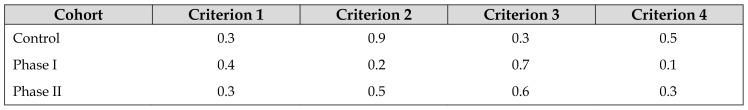
Standard deviation (SD) among evaluators’ rubrics

Cohort	Criterion 1	Criterion 2	Criterion 3	Criterion 4
Control	0.3	0.9	0.3	0.5
Phase I	0.4	0.2	0.7	0.1
Phase II	0.3	0.5	0.6	0.3

Scores for each of the DEBUT criteria were based on an average of each of the evaluators’ individual scores. We used GraphPad InStat 3 to run a series of statistical tests to check for significance among the student performance evaluation criteria, as well as in their citation patterns. We used one-way analysis of variance (ANOVA) to check for statistical significance across all three cohorts, as well as Tukey-Kramer multiple comparisons tests to check for differences among each of the groups. Because ANOVA assumes that the groups have identical SDs, we also tested this assumption using Bartlett’s test. When comparing individual cohort performance across individual criteria, we used an unpaired *t*-test with Welch correction.

## RESULTS

**Figure 1:**
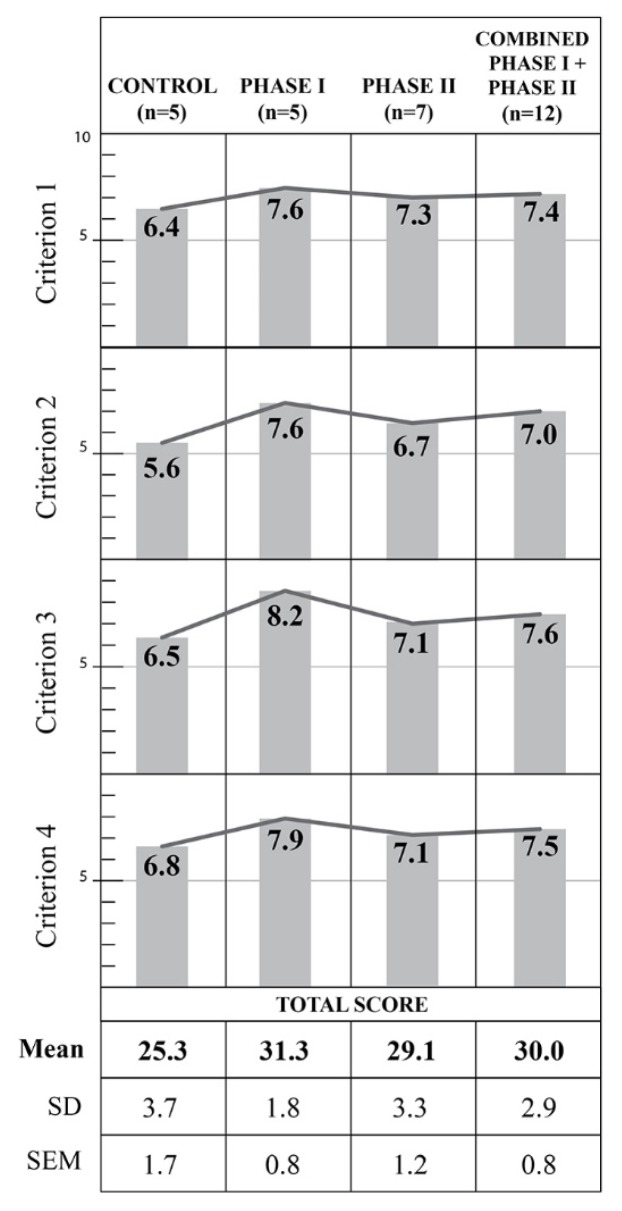
Average student performance for each DEBUT criterion SD=standard deviation; SEM=standard error of the mean.

Average total scores were calculated and are reported along with SD and standard error of the mean (SEM) in Figure 1. ANOVA showed a significant effect of group on total score (F(2, 14)=4.873, *p*=0.0248). Subsequent Tukey-Kramer multiple comparisons tests—where if *q* is greater than 3.702, then the *p* value is less than 0.05—showed significant difference between control and Phase I groups (mean difference [MD]=−6.0, *q*=4.352, 95% confidence interval (CI): −11.104, −0.8960), but no significant differences were found between control and the Phase II groups (MD=−3.8, *q*=2.977, 95% CI: −8.525, 0.9254) or between the Phase I and Phase II groups (MD=2.2, *q*=1.724, 95% CI: −2.525, 6.925). Bartlett’s test showed no significant difference in SD among groups (χ^2^=1.845, *p*=0.3975).

We also compared total score between the control group and the 2 experimental groups combined (Phase I + Phase II), using an unpaired *t*-test with Welch correction, which showed that the combined Phase I + Phase II group had a significantly higher total score than the control group (*t*(6)=2.534, *p*=0.0444, 95% CI: 0.1623, 9.238). We also examined differences in scores at the individual criterion level between the control and combined Phase I + Phase II groups, using unpaired *t*-tests with Welch correction. We found that criterion 2 scores were significantly higher in the combined Phase I + Phase II group than in the control group (*t*(8)=3.230, *p*=0.0121, 95% CI: 0.4578, 2.742). Criterion 3 scores were also significantly higher in the combined Phase I + Phase II group than in the control group (*t*(9)=2.393, *p*=0.0404, 95% CI: 0.060, 2.140). There were no significant group differences in criterion 1 or 4 scores. Full processed student performance data and analysis are available online [32].

The prose and images included in students’ final research assignments across all 3 cohorts showed extensive use of secondary information sources (Table 4); however, ANOVA showed no significant difference in the number of citations among groups (F(2, 14)=0.1251, *p*=0.8834). When evaluating the relationship between the number of citations and total score on the final assignment using a Pearson’s correlation coefficient, we found only a weak positive correlation that did not reach statistical significance (*r*=0.294, *p*=0.25). Full processed student citation data and analysis are available online [33].

**Table 4:**
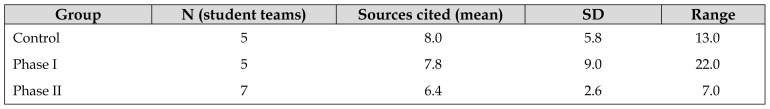
Student citation patterns in design project documentation

Group	N (student teams)	Sources cited (mean)	SD	Range
Control	5	8.0	5.8	13.0
Phase I	5	7.8	9.0	22.0
Phase II	7	6.4	2.6	7.0

## DISCUSSION

These results support the hypothesis that instructors can use information literacy training to introduce health sciences students to the complexities of the economic landscape of health care and to prepare students to evaluate this landscape more effectively. These results align with studies that have previously evaluated the effectiveness of clinical evidence-based practice training programs for health sciences students [34, 35]. The improvement in performance on criterion 2 particularly supports this conclusion, as this criterion measures how well the teams’ projects addressed issues such as market potential, economic feasibility, and patentability. While others have advocated on behalf of teaching students in the sciences how to search for intellectual property information like patents, few of these studies have formally evaluated the effectiveness of this training, which makes comparing these results challenging [36–39].

The improvement in performance on criterion 3 is also noteworthy, as this criterion most closely measures students’ creativity and critical thinking. These findings align with previous studies reporting that information literacy training improves students’ critical thinking skills [40, 41]. We were not surprised that this training did not have a measurable effect on student performance on criteria 1 and 4, as these criteria measure students’ clinical reasoning and engineering design skills. These skill sets, which take years to develop, fall outside the scope of what information literacy training can be expected to impact.

These results did not support our hypothesis that health care economics information literacy training that includes active learning exercises would improve performance over training delivered via lecture, as we did not find a significant improvement in performance between Phase I and Phase II. This result was surprising, as it contradicted several landmark studies in education research that have found that integrating active learning increases student performance in science, technology, engineering, and mathematics undergraduate courses [42, 43]. More focused studies on information literacy instruction for engineers and evidence-based practice training for health sciences students also corroborated the effectiveness of active learning [44, 45]. These data might suggest a point of diminishing returns where additional information literacy training no longer provides substantial extra benefits in increased student learning. For these specific topics, introducing students to issues such as the FDA regulatory environment and Current Procedural Terminology codes via lecture and discussion may provide the same benefits as hands-on exercises. However, this result so starkly contrasts with consensus findings on the benefits of active learning that it may suggest the need for additional studies employing larger samples and randomized assignment.

These results did not support our hypothesis that specialized information literacy training would increase the extent of students’ information use. This finding aligned with some previous studies, which reported that classes that received library instruction often cited nearly the same number of citations as classes that did not receive library instruction [46]. This finding suggests that for instructors who are looking to increase the extent of information that students cite, training on the importance of citations for establishing credibility may not be enough; rather, assignment descriptions that explicitly require the inclusion of a specific number of references may be necessary to direct student behaviors [47]. However, the lack of statistically significant correlation between number of citations and total score in our study suggests that instructors should reconsider assignment designs that require students to cite an arbitrary number of citations.

These results corroborate other findings in the literature that likewise have found little or no correlation between quantity of citations and student performance [48, 49]. Future work examining students’ information-seeking behavior could design a method for evaluating the quality rather than the quantity of sources cited by students. However, attempting to interpret quality of information from a reference list requires a number of assumptions that can negatively impact the validity and generalizability of results [50, 51]. Another possibility is that the design teams’ final research assignments might not be the ideal artifacts for assessing the extent of students’ information use. Assignments completed earlier in the design process, in which students provided summaries of alternative treatment options; current and previous intellectual property; and competitive business landscapes might provide more clear insights into their utilization of secondary information sources than their final assignments, which were largely concerned with describing their own design projects and prototypes.

Because this study used a convenience sample of students who opted into this study, this cohort study does not reflect a true random sample of the full student population. While the relatively prescriptive and sequenced BME undergraduate curriculum ensures that students who enter these courses have completed the same prerequisite courses and have similar credit hours, this study design does not control for variances in ability or motivation in the general student population. As such, the convenience sample represented in this study could be subjected to selection bias, as high-performing teams might have been more likely to opt into the study than low-performing teams.

Students’ ability to find relevant information might also have been impacted by their topic selections, as more novel devices might have less relevant information available (e.g., fewer existing engineering standards, less well-defined regulatory and reimbursement pathways, etc.). Future work in this area could consider randomly assigning students into different groups to increase internal validity as well as recruiting additional student teams to increase the power of the statistical analyses. Additional areas of inquiry could include reviewing different artifacts of student learning and comparing those findings to this analysis of their final design documentation or applying a similar instructional intervention to students who are in affiliated academic programs that intersect with medical entrepreneurship, including medicine, health administration, design, business, and other engineering disciplines.

These results suggest that information literacy training can be used as a method for introducing undergraduate health sciences students to concepts related to medical entrepreneurship and the medical device ecosystem, including the FDA regulatory environment, intellectual property, and medical billing and reimbursement structures. While this study examined undergraduate BME students in particular, these skills may become increasingly important to more health sciences students in the coming years; for example, a handful of allopathic medical schools have already integrated innovation and entrepreneurship into their curricula [52]. As health sciences programs invest more of their students’ curricular hours into innovation and entrepreneurship, it may be necessary for health sciences librarians to gain the expertise needed to help students and faculty navigate the medical entrepreneurship life cycle and to develop programs to support these initiatives [53].

## SUPPLEMENTAL FILES

Appendix A Sources of information for designing medical devices

Appendix B BME 451 Literature searching worksheet: planning your literature search

Appendix C BME senior design DEBUT rubric
