# Supplementary material for: Using information literacy to teach medical entrepreneurship and health care economics
Source: J Med Libr Assoc. 2019 Apr 1;107(2):163–71. doi: 10.5195/jmla.2019.577 (PMC6466497; doi:10.5195/jmla.2019.577)
Supplement: Appendix A [file jmla-107-163-s001.pdf]

JMLA Journal of the Medical Library Association

Supplemental content to
J Med Libr Assoc. Apr;107(2):dx.doi.org/10.5195/jmla.2019.577
www.jmla.mlanet.org
© Carroll, Hallman, Umstead, McCall, DiMeo 2019

Using information literacy to teach medical entrepreneurship and health care economics

Alexander J. Carroll, MSLS, AHIP; Shelby J. Hallman, MSLIS; Kelly A. Umstead, MS, MID; James McCall; Andrew J. DiMeo, PhD

APPENDIX A

Sources of information for designing medical devices

For each of the prompts, select from the options below a source of information that would be the best starting point for searching. In a sentence, explain your selection and a database you could use to find this type of information. You may work with the students seated near you.

Epidemiology data	Standards
Journal articles	Market and legal information
Patents	US Food and Drug Administration (FDA) regulatory information
Company information	Medical device reimbursement information

In order to justify designing an improved angiographic catheter, I want to find information on the prevalence of coronary heart disease in the United States. Where can I find this kind of information?

I want to see whether any X-ray machines currently on the market have been recalled for endangering patients or medical providers. Where can I find this kind of information?

To determine whether a new medical device currently under development is worth bringing to market, I want to examine the feasibility of getting insurance coverage for this device. Where can I find this kind of information?

I want to find current best practices for designing an implantable pacemaker that has optimal biocompatibility. Where can I find this kind of information?

I want to see if there is any current outstanding litigation or if there has been any previous litigation filed against manufacturers of intrauterine devices. Where can I find this kind of information?
