# Supplementary material for: Using information literacy to teach medical entrepreneurship and health care economics
Source: J Med Libr Assoc. 2019 Apr 1;107(2):163–71. doi: 10.5195/jmla.2019.577 (PMC6466497; doi:10.5195/jmla.2019.577)
Supplement: Appendix B [file jmla-107-163-s002.pdf]

JMLA Journal of the Medical Library Association

SC

Supplemental content to
J Med Libr Assoc. Apr;107(2):dx.doi.org/10.5195/jmla.2019.577
www.jmla.mlanet.org
© Carroll, Hallman, Umstead, McCall, DiMeo 2019

Using information literacy to teach medical entrepreneurship and health care economics

Alexander J. Carroll, MSLS, AHIP; Shelby J. Hallman, MSLIS; Kelly A. Umstead, MS, MID; James McCall; Andrew J. DiMeo, PhD

APPENDIX B

BME 451 Literature searching worksheet: planning your literature search

<table>
<tr><td colspan="4">Step 1: What is your project topic?</td></tr>
<tr><td colspan="4"></td></tr>
<tr><td colspan="4">Step 2: Using PICO, identify the main concepts in your topic.</td></tr>
<tr><td>Patient</td><td>Intervention</td><td>Comparison</td><td>Outcome</td></tr>
<tr><td colspan="4">Step 3: Create lists of words or phrases that describe each of your concepts above. Take into account variations in terminology, synonyms, alternate terms, and broader and narrower terms.</td></tr>
<tr><td>Synonyms for patient</td><td>Synonyms for intervention</td><td>Synonyms for comparison</td><td>Synonyms for outcome</td></tr>
<tr><td></td><td></td><td></td><td></td></tr>
<tr><td colspan="4">Step 4: To turn this into a logical search statement, connect the terms in each box with ORs (broadening the search) and those in different columns with ANDs (narrowing the search).</td></tr>
<tr><td colspan="4"></td></tr>
</table>

JMLA Journal of the Medical Library Association

Supplemental content to
J Med Libr Assoc. Apr;107(2):dx.doi.org/10.5195/jmla.2019.577
www.jmla.mlanet.org
© Carroll, Hallman, Umstead, McCall, DiMeo 2019

Tracking your literature searches:

Date	Database	Search statement/terms	Search results/comments
