# Supplementary material for: Using information literacy to teach medical entrepreneurship and health care economics
Source: J Med Libr Assoc. 2019 Apr 1;107(2):163–71. doi: 10.5195/jmla.2019.577 (PMC6466497; doi:10.5195/jmla.2019.577)
Supplement: Appendix C [file jmla-107-163-s003.pdf]

JMLA Journal of the Medical Library Association

SC

Supplemental content to
J Med Libr Assoc. Apr;107(2):dx.doi.org/10.5195/jmla.2019.577
www.jmla.mlanet.org
© Carroll, Hallman, Umstead, McCall, DiMeo 2019

Using information literacy to teach medical entrepreneurship and health care economics

Alexander J. Carroll, MSLS, AHIP; Shelby J. Hallman, MSLIS; Kelly A. Umstead, MS, MID; James McCall; Andrew J. DiMeo, PhD

APPENDIX C

BME senior design DEBUT rubric

Team name: ______________________________________

Design by Biomedical Undergraduate Teams (DEBUT) criteria	Skill set	Score			
		Exceptional (9–10 points)	Satisfactory (6–8 points)	Emerging (4–5 points)	Benchmark (0–3 points)
Does the entry address an important problem or a critical barrier to progress in clinical care or research?	Justifies the problem addressed by explaining the impact on potential users and clinical care.				
How likely is it that the entry will exert a sustained, powerful influence on the problem and medical field addressed?	Evaluates the design concepts for market potential, economic feasibility, and patentability.				
Does the entry utilize novel theoretical concepts, approaches or methodologies, or instrumentation?	Designs the product as a creative response to a need, the functionality of which is driven by people.				
Has evidence been provided (in the form of results, graphs, photographs, films, etc.) that a working prototype has been achieved?	Applies engineering knowledge and skills to build a working prototype.				

Total points: ____________

JMLA Journal of the Medical Library Association

Supplemental content to
J Med Libr Assoc. Apr;107(2):dx.doi.org/10.5195/jmla.2019.577
www.jmla.mlanet.org
© Carroll, Hallman, Umstead, McCall, DiMeo 2019

Adapted from:

- Association of American Colleges & Universities. Information literacy VALUE rubric [Internet]. The Association; 2013 [cited 17 Jun 2014]. <http://www.aacu.org/value/rubrics/InformationLiteracy.cfm>.
- LaBanca F. The 21st-century oral presentation tool bag. Sci Teach. 2013;78(7):51–5.
- VentureWell. DEBUT guidelines [Internet]. VentureWell; 21 Jan 2015[cited 18 Oct 2017]. <https://venturewell.org/guidelines/>.
